# COVID-19: Considerations for Children and Families During the Pandemic

**DOI:** 10.3389/fped.2020.600721

**Published:** 2021-01-14

**Authors:** Binzhi Tang, Didarul Alam, Mejbah Uddin Rakib, Maojun Li

**Affiliations:** ^1^Department of Pediatrics, Sichuan Academy of Medical Sciences and Sichuan Provincial People's Hospital, Chengdu, China; ^2^Department of Pediatrics, Clinical College of University of Electronic Science and Technology of China, Chengdu, China; ^3^The Clinical Medical College of Southwest Medical University, Luzhou, China

**Keywords:** COVID-19, considerations, children, families, pandemic

## Abstract

Coronavirus disease 2019 (COVID-19), a fatal virus caused by severe acute respiratory syndrome coronavirus 2 (SARS-CoV-2), has become a pandemic across the world. Despite early concerns, children appear to be less susceptible to acquiring SARS-CoV-2 and manifest minor clinical symptoms compared with adults. However, there still exists a risk of physical and psychological health problems in children and their families. In this review, we summarize the existing information about the mechanism of SARS-CoV-2 infection, the epidemiology of COVID-19, and the clinical manifestations, treatments, and further considerations regarding COVID-19 in children.

## Introduction

In December 2019, Wuhan received worldwide attention by reporting the first case of COVID-19 in China (1). Although the source of COVID-19 has not yet been clearly defined, the patient's history was linked to a seafood market in Wuhan, China during the early period of the outbreak. Animal to human transmission followed by human to human transmission has been presumed, though not verified (1). The outbreak of COVID-19 spread widely and rapidly and has now affected more than 205 countries. The World Health Organization (WHO) declared COVID-19 as a pandemic on March 11, 2020. Earlier investigations suggested numerous possible COVID-19 transmission routes including respiratory droplets and aerosols, close contact, and surface areas exposed to SARS-CoV-2 (2). In addition, intrauterine and transplacental transmission have been reported (3). Children do not appear to transmit SARS-CoV-2 as readily as adults (4) and usually have milder symptoms (5, 6). This mysterious coronavirus is confusing scientists, public health specialists, and global populations by demonstrating its variable characteristics.

Patients with mild to moderate COVID-19 symptomatology typically demonstrate flu-like symptoms such as fever, cough, dyspnea, and myalgia, most of which are generally not of great severity and self-resolve with time. In contrast, clinical complications such as ARDS, sepsis, multiple organ failure (MODS), and coagulopathy, etc., are found in severe cases and translate to a high mortality and morbidity rate (7).

Clinicians apply many treatment protocols to treat patients infected with SARS-CoV-2 and a series of trials are ongoing. Among treatment protocols, pharmacologic management typically consists of remdesivir (8), lopinavir/ritonavir (9), interferon beta-1α (9), favipiravir (10), corticosteroids (11, 12), and ribavirin (13). Initial results demonstrated that remdesivir (8), and corticosteroids (11, 12) were likely to play a leading role in the treatment of COVID-19. Unfortunately, potential side/adverse effect such as anemia, acute kidney injury, hyperglycemia, nausea, acute respiratory failure, elevated transaminases, jaundice, and constipation have been reported in remdesivir (14). Although the WHO welcomed the clinical use of dexamethasone, there does not appear sufficient safety and efficacy data to support corticosteroid administration in neonates and pregnant women (15). Besides pharmacologic interventions, supplemental and supportive therapies appear to have stronger data supporting use. For example, both plasma therapy and oxygen therapy are helpful in broad patient populations (16). Without specific pharmacologic treatments or a current vaccine, patients of all ages are susceptible to the consequences of this deadly virus. Fortunately, there are now at least five phase III clinical trials for SARS-CoV-2 vaccines (17). However, further study is necessary before widespread vaccine dissemination to global populations is possible.

## Mechanisms of SARS-CoV-2 Infection

**Figure 1:**
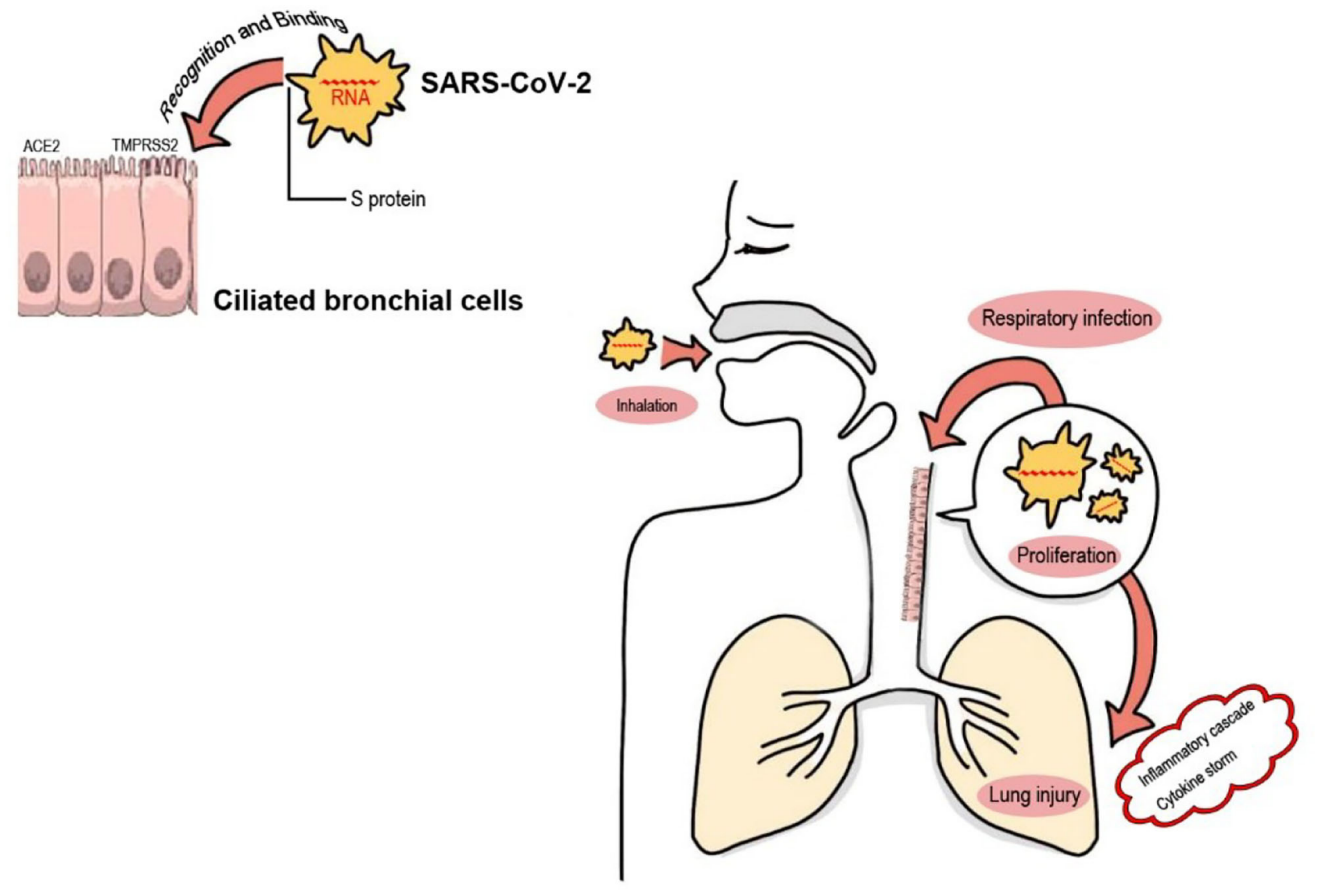
SARS-CoV-2 infection via the airway. During inhalation, SARS-CoV-2 in the air reaches and colonizes in the bronchioles or alveoli. Spike-like protein S on the surface of the SARS-CoV-2 virus serves as a ligand, which recognizes and binds to its specific receptors (the ACE2 and the TMPRSS2 receptors) on human host cell (ciliated bronchial cell) membranes with a high affinity. This recognition and interaction between host cell membranes and viral surface molecules permit SARS-CoV-2 virus entrance into target cells resulting in intracellular proliferation of viral copies. This pathogenic process of respiratory infection leads to the activation of both extracellular and intracellular cytokines, and the subsequent “inflammatory cascade” and “cytokine storm syndromes” of COVID-19, which finally result in pulmonary or other tissue injury (20), or even a rare but lethal post-COVID-19 complication termed “multisystem inflammatory syndrome in children (MIS-C)” (21).

SARS-CoV-2, a new member of the Coronaviridae family, is a single stranded positive RNA virus. Up to now, coronavirus strains Human coronavirus-NL63 (HCoV-NL63), HCoV-HKU1, HCoV-229E, and HCoV-OC43 are known to cause the “common cold” in humans (18). SARS-CoV-2 and the severe acute respiratory syndrome coronavirus (SARS-CoV) have similar homologies with ~88% of the genomic materials which cause COVID-19 (19). SARS-CoV and SARS-CoV-2 are similar to most other virus in that the recognition and interaction between host cell membranes and viral surface molecules initiate the viral infection. As shown in Figure 1 and Table 1. SARS-CoV-2 uses a spike S protein which has two subunits, S1 and S2, to identify the angiotensin-converting enzyme 2 (ACE2) and the type 2 transmembrane serine protease (TMPRSS2) receptors (31). Both host cell receptors allow viral penetration in ciliated bronchial cells and facilitate SARS-CoV-2 entrance into the human host cells (31). The spike protein of SARS-CoV-2 found in 2019 shows a 10–20 fold higher receptor-binding affinity than SARS-CoV and MERS-CoV (31).

**Table 1:** Comparable characteristics between COVID-19/ SARS-CoV-2 infected adults and children.

**COVID-19**	**Adults**	**Children**
Epidemiology	To date (December 5 2020), Worldwide more than 65 million have been infected and 1.5 million have died according to the WHO (22).	About 2, 12, and 1.2% Children infected in China, USA, and Italy, respectively (23–25).
Clinical manifestations	May be asymptomatic. Fever, cough, and shortness of breath are most common. Other symptoms include headache, myalgia, sore throat, and digestive symptoms. High fever, chest tightness, ARDS, and MODS can be seen in severe pneumonia (7).	Mild or asymptomatic clinical symptoms in most cases. Fever, cough, and sore throat are most common. Myalgia, fatigue, digestive symptoms, and rhinorrhea are relatively rare (26, 27). The decrease of lymphocyte count in children is usually milder than in adult patients (27). Serious condition characterized by MIS-C or Kawasaki-like disease (21).
Transmission mode/route	By respiratory droplets, close contact, surface contact, and secretion (eye, nose).	In addition to general transmission routes in adults, intrauterine transmission has also been proven in several cases (3). Although SARS-CoV-2 has been detected in the feces of COVID-19 children (28), there is no clear evidence of fecal-oral transmission.
Prevention	Isolation of patient. Social distancing, using a mask and hand sanitizer, and washing hands. The most effective is undoubtedly the specific vaccine now in research.	Similar to general prevention measures in adults. Additionally, for newborns delivered by SARS-CoV-2 positive mothers, initiate newborn prophylaxis immediately and undertake preventive precautions when breastfeeding (29).
Therapy	Supportive and symptomatic therapies in mild to moderate cases. Corticosteroids and potential antiviral drugs such as remdesivir, favipiravir, INFα, and lopnavir/ritonavir are alternatives (8–14). Mechanical ventilation and/or other advanced life supports like CRRT or ECMO may be necessary in severe cases.	Supportive and symptomatic therapies in mild to moderate cases. Corticosteroids and potential antiviral drugs are used with caution (15). Seldom use of advanced life supports. COVID-19 patients, once diagnosed with concurrent KD should be treated with intravenous immunoglobulin (IVIG) and high-dose aspirin (ASA) (30).
Prognosis	Depends on the viral load and virulence, human immunity, and treatment.	Generally, good.

SARS-CoV-2 may present super antigenic fragments that could bind to the αβT-cell receptors (αβTCRs) and induce an inflammatory response. Pathogens with amino acid sequences and protein structures similar to SARS-CoV-2 can also activate an αβTCRs-induced inflammatory response (21). A case in point is staphylococcal enterotoxins B (SEB) toxin, which is known to be involved in toxic shock syndrome (TSS) (21). Besides, specific strains of coronavirus have also been associated with Kawasaki disease (KD), and the activation of pro-inflammatory cytokines in MIS-C patients overlapped with laboratory findings in both KD and patients with COVID-19 (32). Moreover, the cytokine storm pattern in MIS-C includes implicated macrophage activating syndrome (MAS), which is also seen in KD (32). Although the actual molecular mechanism of MIS-C, TSS, and KD are still under investigation, such immunological similarities would in part explain the analog manifestations and the inflammatory responses described in these syndromes (21).

## Investigation in Pediatric Epidemiology

On January 10, 2020 the first pediatric case was reported in Shenzhen, China (33). A report from the Chinese Center for Disease Control and Prevention found that as of mid-February, among the 72,000 Chinese patients infected with SARS-CoV-2, <1% were children under 10 years of age. As of that time, no fatality was reported in children younger than 9 years old (23). In the United States of America (US), the latest data (available as of November 26, 2020) reported a total of 1,337,217 child COVID-19 cases, with children representing 12.0% (1,337,217/11,184,900) of all cases, with an overall rate of 1,777 cases per 100,000 children in the population (24). Of note, a trend of drastically increasing new child COVID-19 cases was reported in the past few weeks (24). In Italy, one of the most affected countries in Europe, 1.2% of children between 0 and 18 years old were infected with SARS-CoV-2 by March 18, 2020 (25). Unfortunately, a multinational study including 409 children from Latin American reported that 23.2% of pediatric COVID-19 patients were diagnosed with MIS-C and 12.7% required admission to a pediatric intensive care unit, indicating a higher incidence of MIS-C and a more serious condition, compared with studies from other areas (34). Therefore, more care for pediatric COVID-19 cases in Latin America or other lower middle income countries (LMICs) are urgently needed. In comparison with the current measures of SARS-CoV-2, the number of pediatric cases was relatively low and no fatality was recorded during the epidemic of SARS and MERS in 2003 (35). What is more, to date, all ages of childhood (ranged from 1 day to 18 years) were reported to be susceptible to SARS-CoV-2 (36).

A recent study published in *JAMA Pediatrics* revealed that children younger than 5 years with mild to moderate COVID-19 carry higher levels of viral genetic material in the nasopharynx compared to older children and adults (37). These findings suggest that younger children transmit the virus as much as other age groups. The ability of younger children to spread COVID-19 may have been under-recognized given the rapid and sustained closure of schools and daycares during the early stages of the pandemic. Interestingly, these children with higher virus loads are less susceptible to SARS-COV-2 infection and mild symptoms compared to adults. The lower risk of symptomatic infection among children appears to be due to age-dependent expressions of *ACE2* which is lower in younger children and increases with age. Lower *ACE2* expression in children relative to adults may help explain why COVID-19 is less prevalent in children (38).

## Clinical Manifestations

Due to its latent period of ~2–14 days and vague symptoms, COVID-19 can be misdiagnosed as other more benign viral processes, especially in the early phase of infection (39). Clinical manifestations of COVID-19 in adults and children are similar and include asymptomatic fever, cough, and shortness of breath. Other symptoms include headache, myalgia, sore throat, or digestive symptoms such as vomiting, nausea, and diarrhea (26). Compared with fever or respiratory symptoms, vomiting and diarrhea were not so common in children (27) and there were no severe clinical gastrointestinal (GI) symptoms in children with COVID-19 infection (40). However, more frequent GI symptoms, as well as high fever, chest tightness, ARDS, or MODS have been found in severe COVID-19 cases (7, 21).

Most studies have focused on COVID-19 in adults, as early data suggested that symptoms tend to be milder in the pediatric population (4). However, since May 2020, there has been a rise in the number of critically ill COVID-19 patients presenting a co-incidence of post infectious MIS-C (32) or common features of KD, especially in Europe and the US (30, 41). Cases of MIS-C and/or Kawasaki-like disease in the setting of COVID-19 infection were usually reported to exhibit clinical symptoms including fever, lymph node enlargement, limbic sparing conjunctivitis, prominent tongue papilla, polymorphous, maculopapular rash, extremity edema, multisystem inflammation or MODS, ARDS, TSS or KD shock syndrome, abdominal pain, and severe cardiac involvement (21, 30, 42). Although the overall post-COVID-19 MIS-C presentation appears to overlap with KD as mentioned above, MIS-C children present with noticeable differences compared to KD, including: older age of presentation, a more profound form of inflammation, more GI manifestation, different laboratory findings such as lymphopenia, thrombocytopenia, elevated troponin, elevated NT-proBNP, elevated D-Dimer, and elevated ferritin, and have a higher propensity toward LV dysfunction and shock (32).

The overlapping clinic features of MIS-C, TSS, and KD may be attributed to their immunological similarities as discussed above: MIS-C, TSS, and KD are all post-infectious immune-mediated diseases and share similar post-infectious systemic immune responses (21). Interestingly, unlike KD, MIS-C seem to have less propensity toward East Asian children, the observation of MIS-C was mostly reported in Europe, the US, and Latin American, suggesting an involvement of specific HLA types in COVID susceptibility.

## Transmission of COVID-19: Mother to Neonates

Available studies have identified rare transmission from mother to fetus via vertical or intrauterine transmission (43). In one of study from China, 19 neonates were born from SARS-CoV-2 positive mothers but none developed SARS-CoV-2 (44). Mothers' breast milk and amniotic fluid were also tested negative for SARS-CoV-2 (44). All neonates were separated from their mothers after birth and were in observation for 14 continuous days (44). No respiratory distress, clinical, radiologic, or hematologic signs related to SARS-CoV-2 were seen during these 14 days of observation (44). These findings provide insufficient evidence of vertical transmission and prenatal complications in mothers infected with SARS-CoV-2. Another study from the US described 29 neonates from SARS-CoV-2 positive mothers and none of the neonates were found to be SARS-CoV-2 positive or had associated symptoms (45). Similarly, nine children born via cesarean section were negative for SARS-CoV-2 and did not develop associated symptoms (43). Although earlier studies suggested a low risk for vertical transmission, a recent study by Vivanti et al. provided confirmatory evidence of transplacental transmission from mother to fetus (3). Zeng et al. described three of 33 neonates from Wuhan Children's Hospital, who had positive results for SARS-CoV-2 following their operative delivery by mothers with confirmed COVID-19 (46).

A synthesis and systematic review of 176 published cases reported recently that 70 and 30% of infections were due to environmental and vertical transmission, respectively, and suggested that a lack of mother-neonate separation from birth was associated with late SARS-CoV-2 infection, while breastfeeding was not (47). However, a population-based prospective national cohort study in the UK supported guidance to avoid the routine separation of mother and baby because neonatal SARS-CoV-2 infection was uncommon and most babies were only mildly affected (48). Moreover, it should be noticed that an excretion of SARS-CoV-2 has been found in COVID-19-infected mothers' breast milk (49), yet breastfeeding does not seem to significantly increase the risk of maternal-fetal transmission. The existence of protective anti-SARS-CoV-2 immunoglobulins in COVID-19-infected mothers' milk is likely to be a major contributor to the lower risk of viral transmission through milk (50). Nevertheless, horizontal transmission from a positive SARS-CoV-2 infected mother to her baby may take place through respiratory droplets or close contact during breastfeeding. Since these newly described studies have yielded conflicting results, larger studies are necessary for verification as well as quantification of the risk to mothers and infants. Currently, a rinse with room temperature water immediately after vaginal delivery and breastfeeding with hygiene precautions are generally recommend for newborns following birth to a mother with perinatal SARS-CoV-2 infection.

## Warning and Caution for Parents

Children, especially infants and young children, may have difficulties in understanding how to keep healthy during the COVID-19 pandemic. Parents have an obligation to explain disease prevention measures to their children and help prevent children acquiring and spreading infectious diseases. For all parents, better knowledge and awareness of COVID-19 and a positive attitude toward the prevention and control of COVID-19 are of great importance for improving their management of physical and psychological health. Parental considerations are briefly summarized below:

1. Large public gatherings are dangerous since it is difficult to socially distance from those nearby. Parents should be particularly cautious in a hospital environment since there may be asymptomatic carriers and patient populations close by that are particularly vulnerable to developing the life threatening consequences of COVID-19.
2. Since SARS-CoV-2 is highly contagious, behavioral precautions, including hand hygiene and mask-wearing, must be repeatedly emphasized. Adhering to self-quarantine and practicing necessary hygienic habits will effectively curtail viral spread.
3. Sometimes it is difficult to distinguish between common cold and COVID-19 symptoms, especially at the early stages of the infection. If there has been a possible recent exposure alongside symptoms like fever, cough, muscle pain, headache, both the children and other family members should go to hospital for a laboratory test of SARS-CoV-2 infection as soon as possible.
4. Be aware of COVID-19 children with a confirmed or suspected co-incidence of other severe clinical conditions such as MIS-C or KD, timely hospitalization and intensive care are necessary, and IVIG is recommended as early as possible, with the aim of preventing MIS-related complications and KD-induced coronary artery aneurysms.
5. Vaccinations would be the easiest, most effective, and fastest preventive strategy available to reduce pediatric communicable diseases and consequently reduce the overflow of children to pediatric services. Therefore, the highest immunization levels, including vaccinations against diphtheria, tetanus, pertussis, hepatitis B, measles, mumps, rubella, meningococcal and pneumococcal vaccination, and especially influenza, are of pivotal importance during the COVID-19 pandemic. Vaccine education is necessary for parents to attain knowledge of each recommended vaccine, and their awareness that vaccination services should focus on not only the pediatric population, but also all the family members (51).
6. Mothers developing COVID-19 during pregnancy can deliver prematurely and may have complicated outcomes (52). Thus, adequate pre-natal care for a pregnant mother during these times is important. If an infant is born to a parent with COVID-19, mask wearing and careful hand hygiene are required when caring for the baby. Guidelines in different countries for managing neonates are conflicting. In China, infants born to mothers with COVID-19 were separated for a minimum of 14 days and until the mother's COVID-19 tests turned negative. Children were not allowed to breast feed if born to a COVID-19 positive mother (52). There was no reliable evidence to support vertical transmission in the beginning stages of the COVID-19 outbreak, yet recently data suggest the potential of transplacental transmission from mother to fetus. Therefore, although there still exists controversy about this type of transmission, it is a viable guideline for effectively protecting vulnerable neonates and their parents from virus transmission.
7. Outbreaks of emerging infectious diseases and consequential quarantine in homes and institutions may impose great psychological burdens to families, and lead to negative social phenomena. School closure, lack of outdoor activity, and aberrant dietary and sleeping habits are likely to disrupt a child's usual lifestyle and can potentially promote distress, anxiety, annoyance, and varied neuropsychiatric manifestations (53). Children fearing stigmatization may deny early clinical symptoms, delay seeking care, and remain in the community undetected, which may aggravate the spread of the infection (54). Parents should exert effort to mitigate the psychosocial ill-effects of COVID-19 on children.
8. Both parents and their children (especially teenagers aged 13–18) have been more exposed to social media and other types of communication technologies since the beginning of social distancing. As social media use is widespread, there is potential for the rapid dissemination of false information which can lead to poor behaviors and/or undue stress and anxiety. Utilization of social media should be limited in children in early development and those with psychological vulnerabilities (55). Communication technologies can also help to support parents and children. Electronic medical consultation (“telemedicine”) could also be utilized for medical evaluations and many age-appropriate educational programs can be found online (51). A case of benefit from communication technologies during the COVID-19 pandemic was summarized by two Bangladeshi intern doctors and their teachers, who instructed them thousands of miles away by web-based communication.
9. Parents are attempting to work remotely, though may be unable to work due to family needs or may find themselves unemployed with uncertainty about how long the pandemic will last. The economic crisis and need for home childcare increases parenting stress, abuse, and violence against children. For those living in low-income and crowded households, these challenges are exacerbated (56).

## Conclusion

In response to the increasing seriousness of COVID-19 as a threat to human health, researchers continue to pursue effective strategies to mitigate the physical and psychological damage due to SARS-CoV-2 infection in children and families. If children or their parents become sick with COVID-19, following guidance from local agencies and the WHO can reduce viral spread and foster a healthy environment for families.

In addition, there are so many overlapping features of KD and pediatric COVID-19 or MIS-C, including epidemiological characteristics, pathogenesis mechanisms, clinical manifestations, and therapeutic strategies. First, KD and COVID-19 are epidemiologically similar: KD is primarily distributed in East Asia, followed by Western Europe, North America, and other LMICs such as Latin America, while COVID-19 was first reported in China and then spread all over the world, of which Western Europe and North America are the worst-hit areas nowadays. Besides, the peak of KD usually occurs in the winter or spring (from January to March) while COVID-19 has been emerging in China since December 2019 and then spread to other countries and regions around the world since February 2020, suggesting a winter-spring seasonality of both KD and COVID-19. Second, as previously discussed, pathogenesis mechanisms involved in KD and MIS-C post-COVID-19 infection are similar: both of them are the result of an excessive immune response to an infectious trigger, which could stimulate the immune system with a strong systemic inflammatory response finally leading to organ damage (42). Third, as described above, KD and MIS-C exhibit similar clinical symptoms. And last, most of the immunomodulatory therapeutic strategies used for KD have been shown to be effective for MIS-C, including IVIG and steroids (21). All of this evidence suggests a connection between pediatric COVID-19/MIS-C and KD. It is necessary that physicians, institutions, policy makers, and families all prepare themselves with enough time to face the difficulties of the increasing cases of these pediatric COVID-19 associated severe diseases in the coming winter.

## Author Contributions

BT, DA, and MR searched the literature and wrote the manuscript. BT designed the figure and table. BT and ML contributed to the critical revision of the article, suggested additional references, and wrote the manuscript. All authors read and approved the final manuscript.

## Conflict of Interest

The authors declare that the research was conducted in the absence of any commercial or financial relationships that could be construed as a potential conflict of interest.
